# Supplementary material for: In silico analysis identifies a putative cell-of-origin for BRAF fusion-positive cerebellar pilocytic astrocytoma
Source: PLoS One. 2020 Nov 18;15(11):e0242521. doi: 10.1371/journal.pone.0242521 (PMC7673500; doi:10.1371/journal.pone.0242521)
Supplement: S1 Data — (PDF) [file pone.0242521.s001.pdf]

Data Supplement 1

This supplement contains *in situ* hybridization images from the Allen Developing Mouse Brain Atlas for each PA-DR gene and formed the basis of the data presented in summary form in Fig. 2A and 2B as well as Supplementary Table 2. Each page contains a representative image for the indicated gene on embryonic day 13.5 or 15.5. Areas of positivity stain black while the cellular counter stain is light brown/yellow.

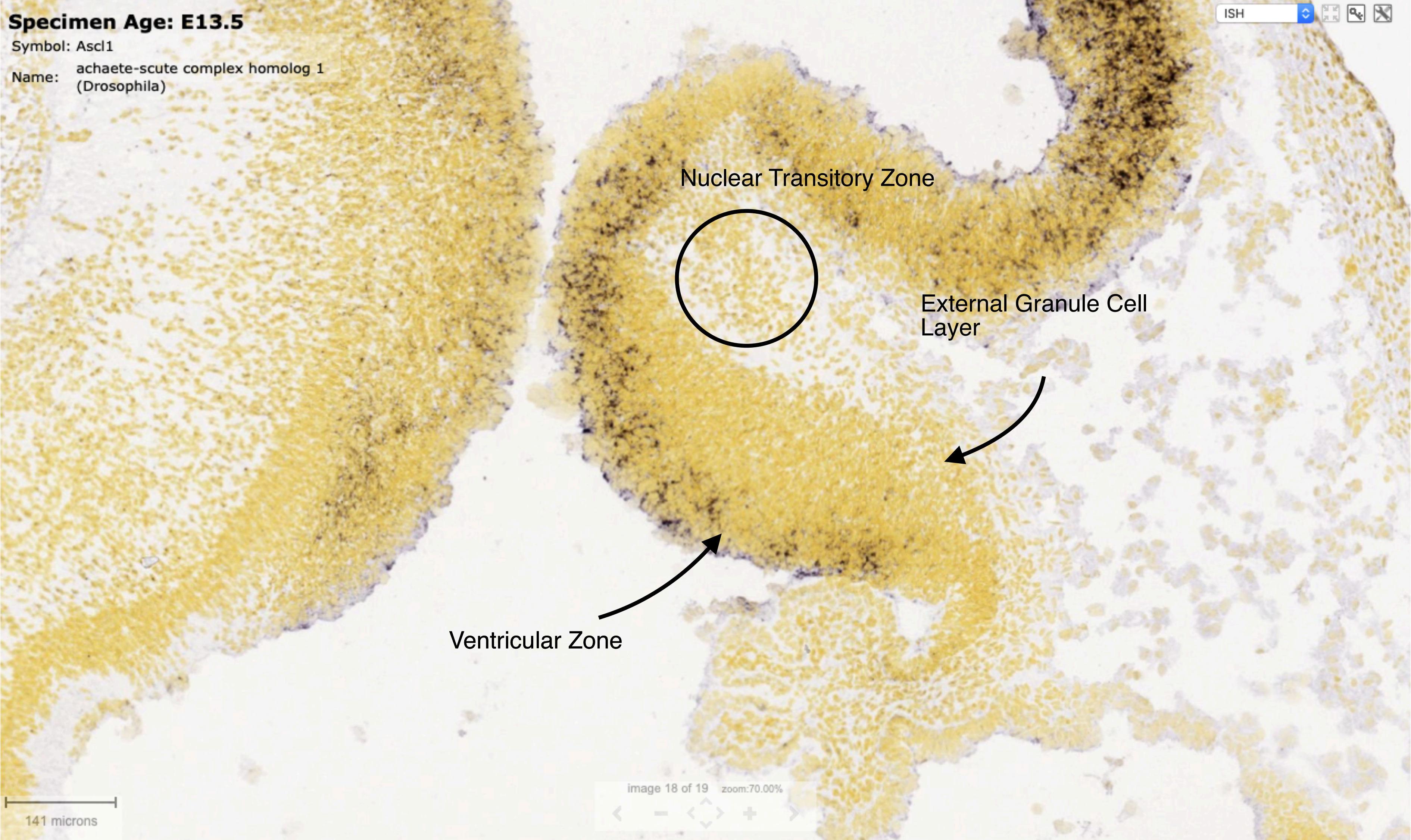
Specimen Age: E13.5
Symbol: Ascl1
Name: achaete-scute complex homolog 1 (Drosophila)
ISH
Nuclear Transitory Zone
External Granule Cell Layer
Ventricular Zone
image 18 of 19 zoom:70.00%
141 microns

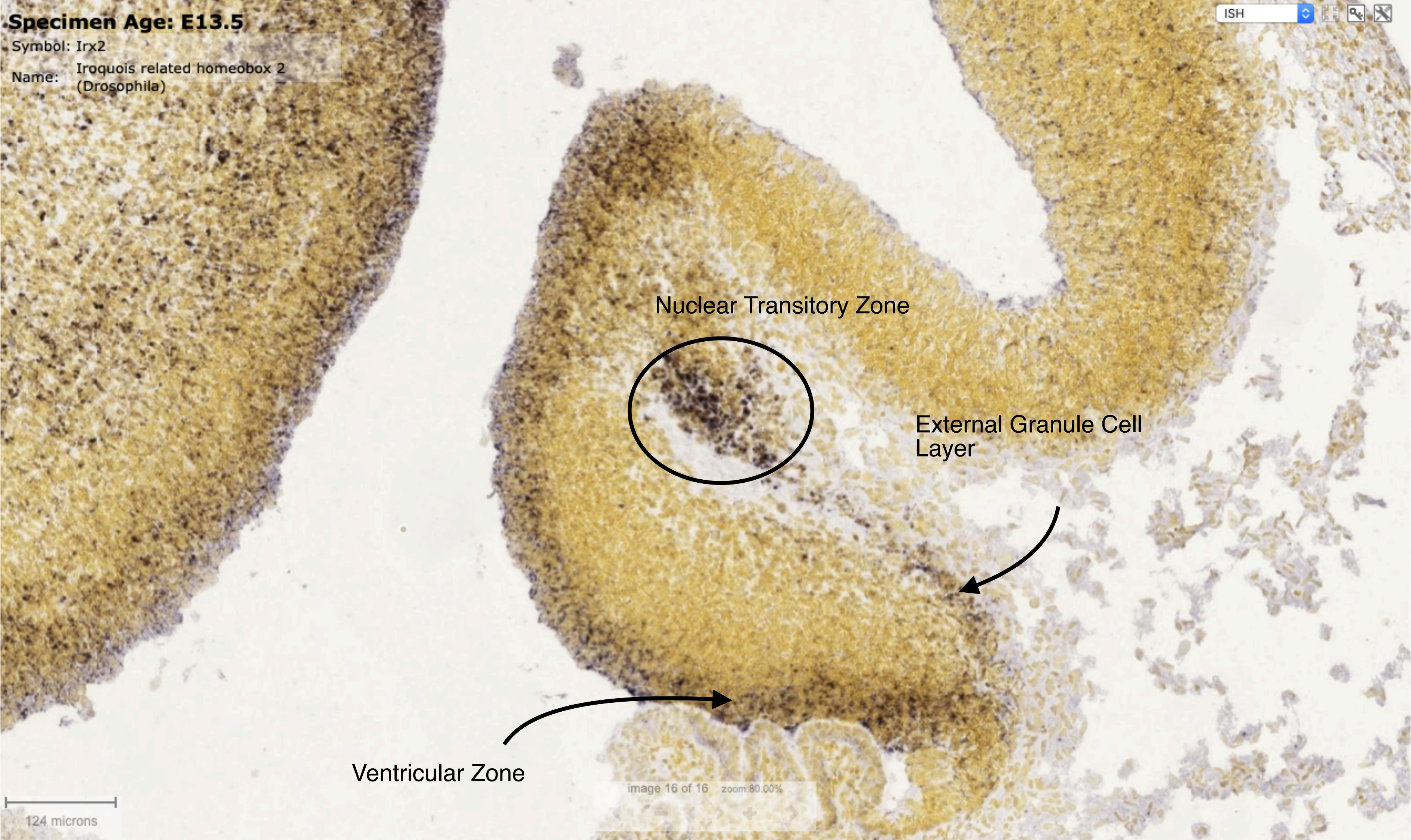
Specimen Age: E13.5
Symbol: Irx2
Name: Iroquois related homeobox 2 (Drosophila)
ISH
Nuclear Transitory Zone
External Granule Cell Layer
Ventricular Zone
image 16 of 16
zoom:80.00%
124 microns

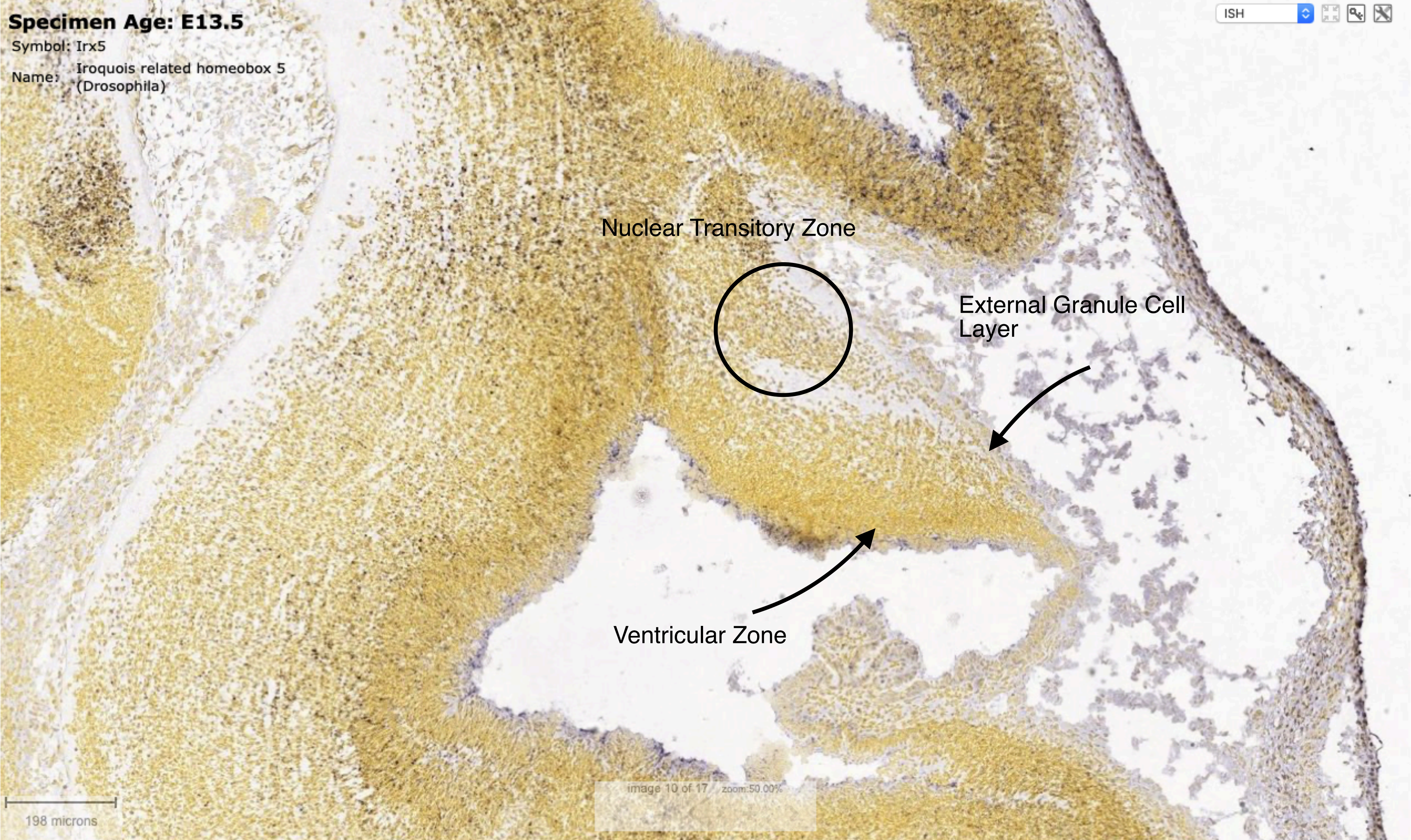
Specimen Age: E13.5
Symbol: Irx5
Name: Iroquois related homeobox 5 (Drosophila)
ISH
Nuclear Transitory Zone
External Granule Cell Layer
Ventricular Zone
image 10 of 17
zoom:50.00%
198 microns

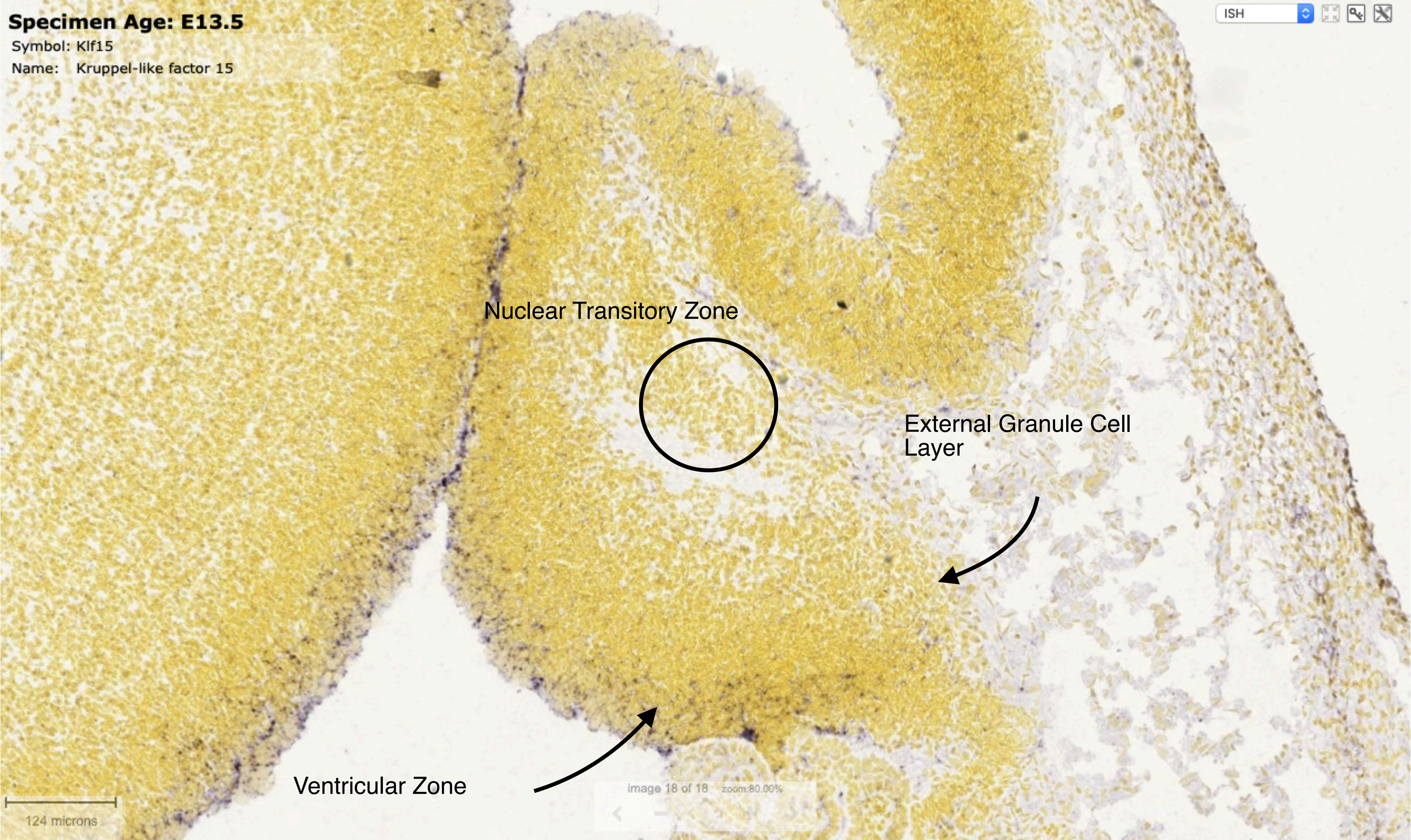
Specimen Age: E13.5
Symbol: Klf15
Name: Kruppel-like factor 15
ISH
Nuclear Transitory Zone
External Granule Cell Layer
Ventricular Zone
image 18 of 18
zoom:80.00%
124 microns

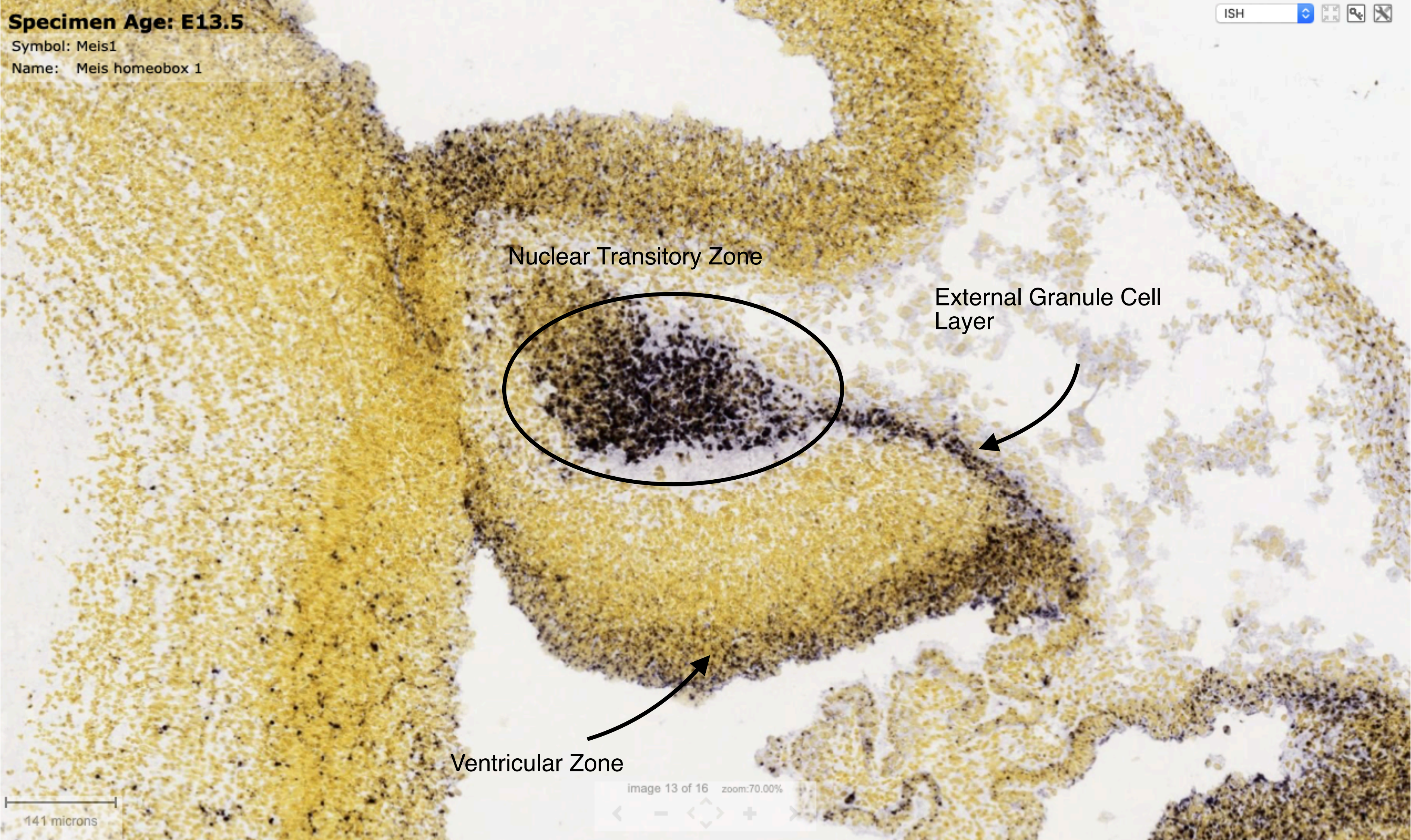

Specimen Age: E13.5
Symbol: Meis1
Name: Meis homeobox 1
ISH
Nuclear Transitory Zone
External Granule Cell Layer
Ventricular Zone
image 13 of 16
zoom:70.00%
141 microns

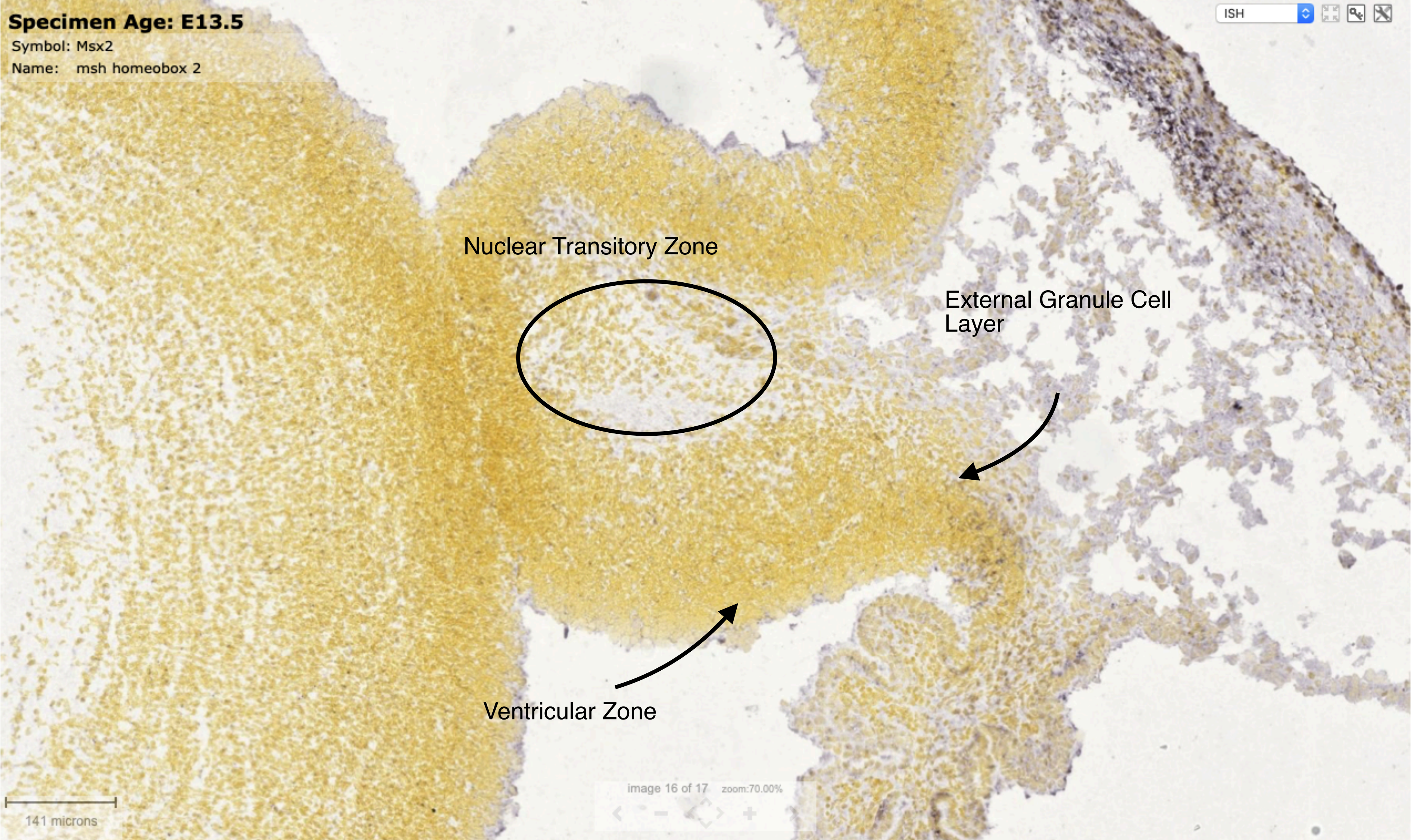
Specimen Age: E13.5
Symbol: Msx2
Name: msh homeobox 2
ISH
Nuclear Transitory Zone
External Granule Cell Layer
Ventricular Zone
image 16 of 17
zoom:70.00%
141 microns

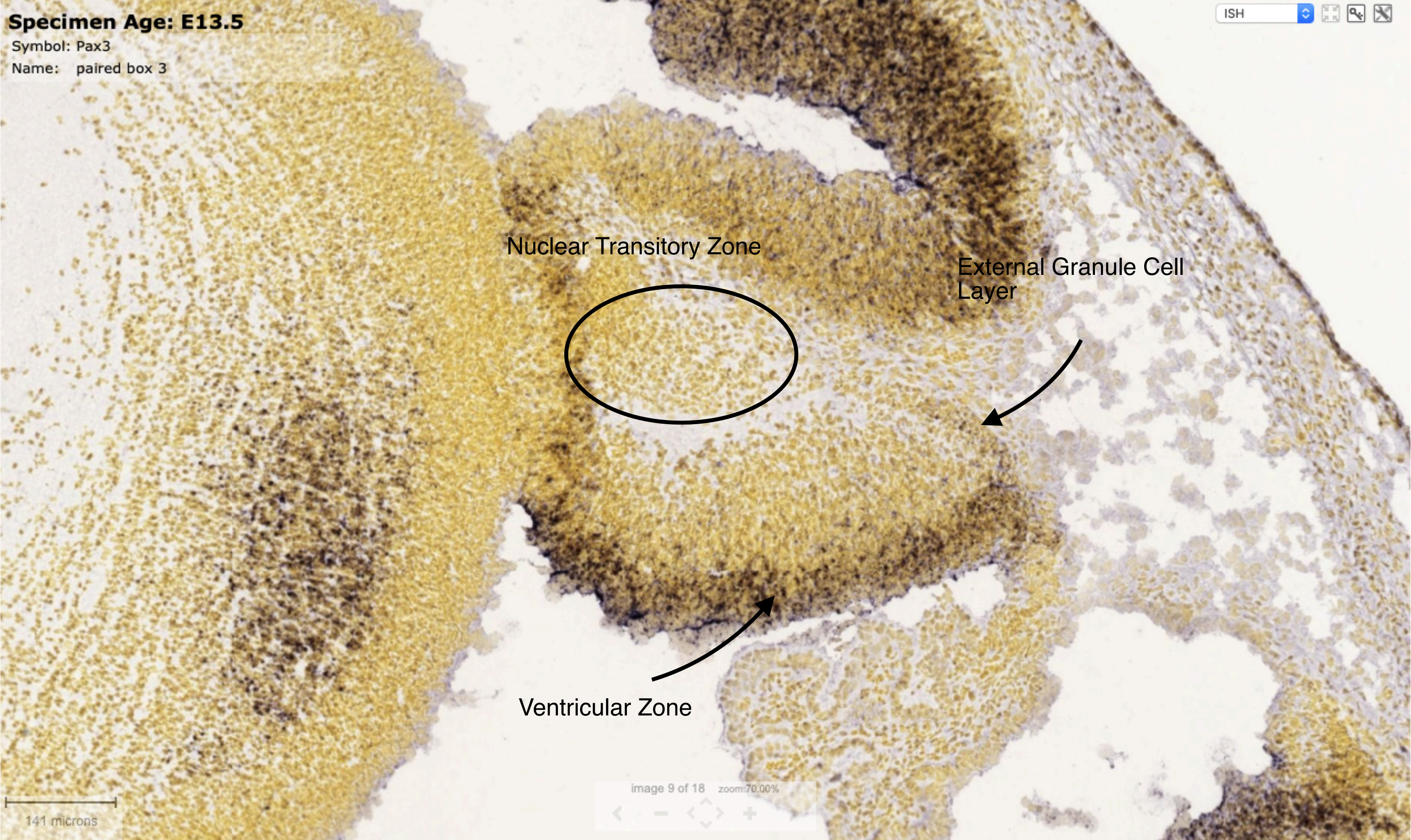
Specimen Age: E13.5
Symbol: Pax3
Name: paired box 3
ISH
Nuclear Transitory Zone
External Granule Cell Layer
Ventricular Zone
image 9 of 18
zoom:70.00%
141 microns

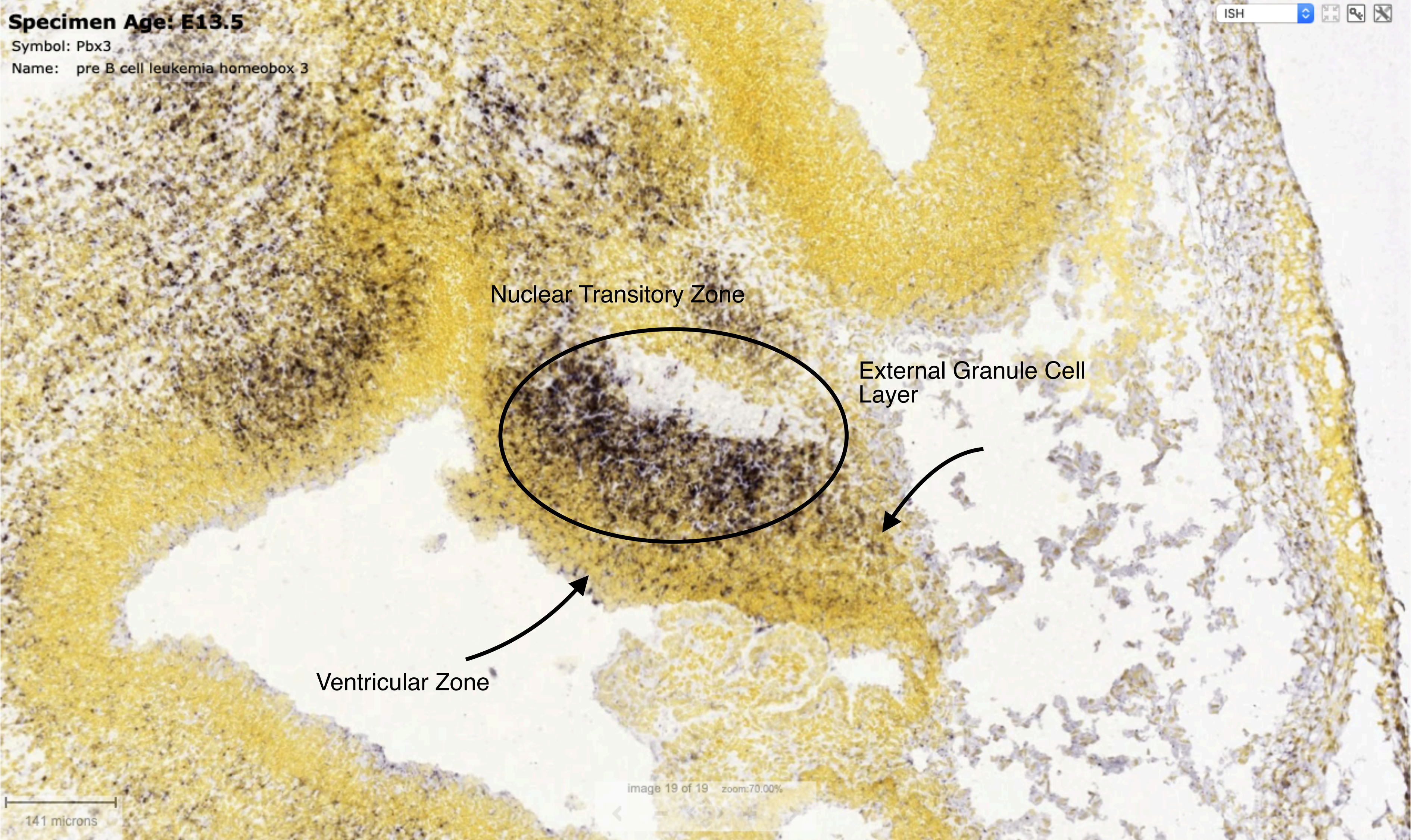
Specimen Age: E13.5
Symbol: Pbx3
Name: pre B cell leukemia homeobox 3
ISH
Nuclear Transitory Zone
External Granule Cell Layer
Ventricular Zone
image 19 of 19
zoom:70.00%
141 microns

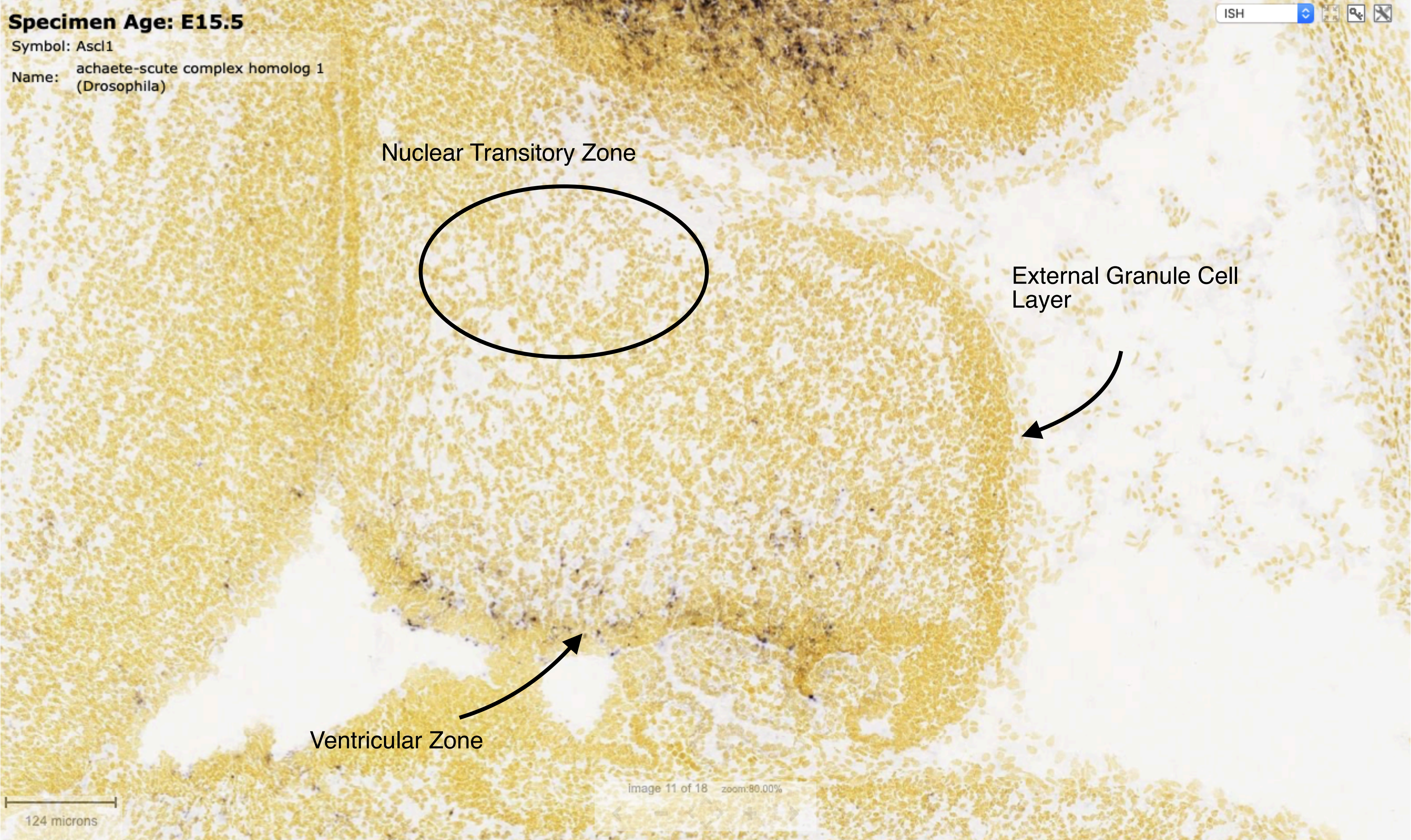
Specimen Age: E15.5
Symbol: Ascl1
Name: achaete-scute complex homolog 1 (Drosophila)
ISH
Nuclear Transitory Zone
External Granule Cell Layer
Ventricular Zone
image 11 of 18
124 microns

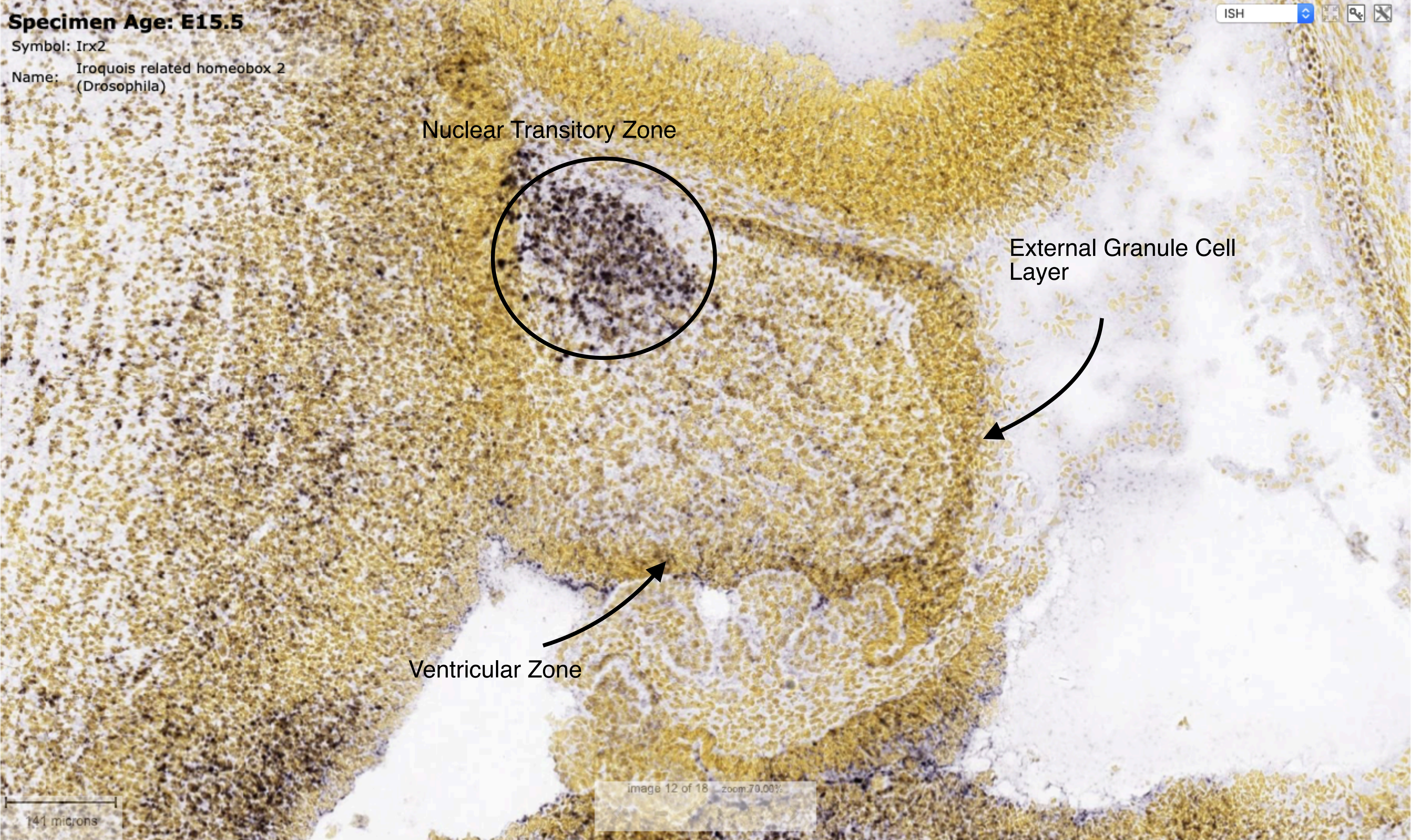

Specimen Age: E15.5
Symbol: Irx2
Name: Iroquois related homeobox 2 (Drosophila)
ISH
Nuclear Transitory Zone
External Granule Cell Layer
Ventricular Zone
image 12 of 18
zoom 70.00%
141 microns

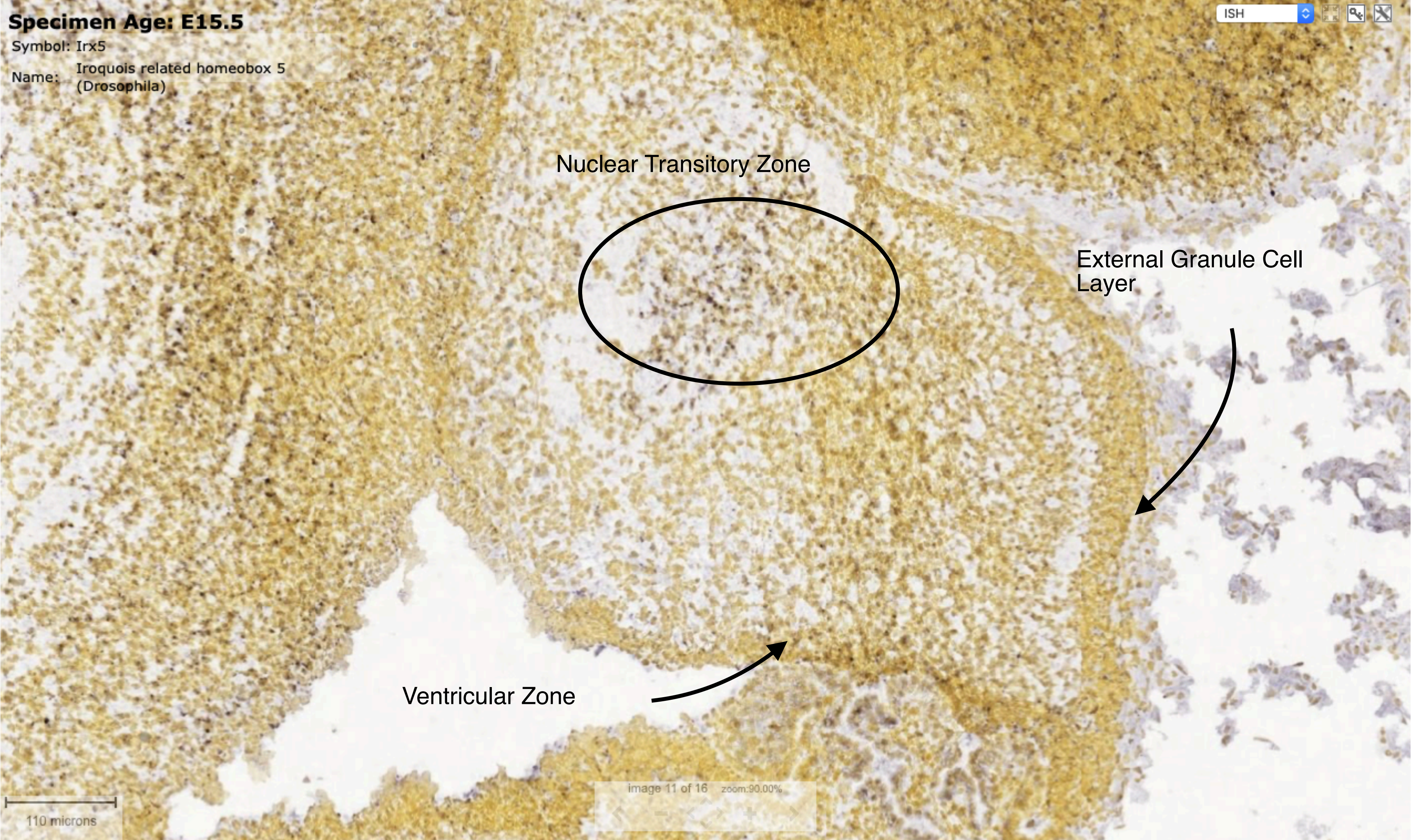
Specimen Age: E15.5
Symbol: Irx5
Name: Iroquois related homeobox 5 (Drosophila)
ISH
Nuclear Transitory Zone
External Granule Cell Layer
Ventricular Zone
image 11 of 16 zoom:90.00%
110 microns

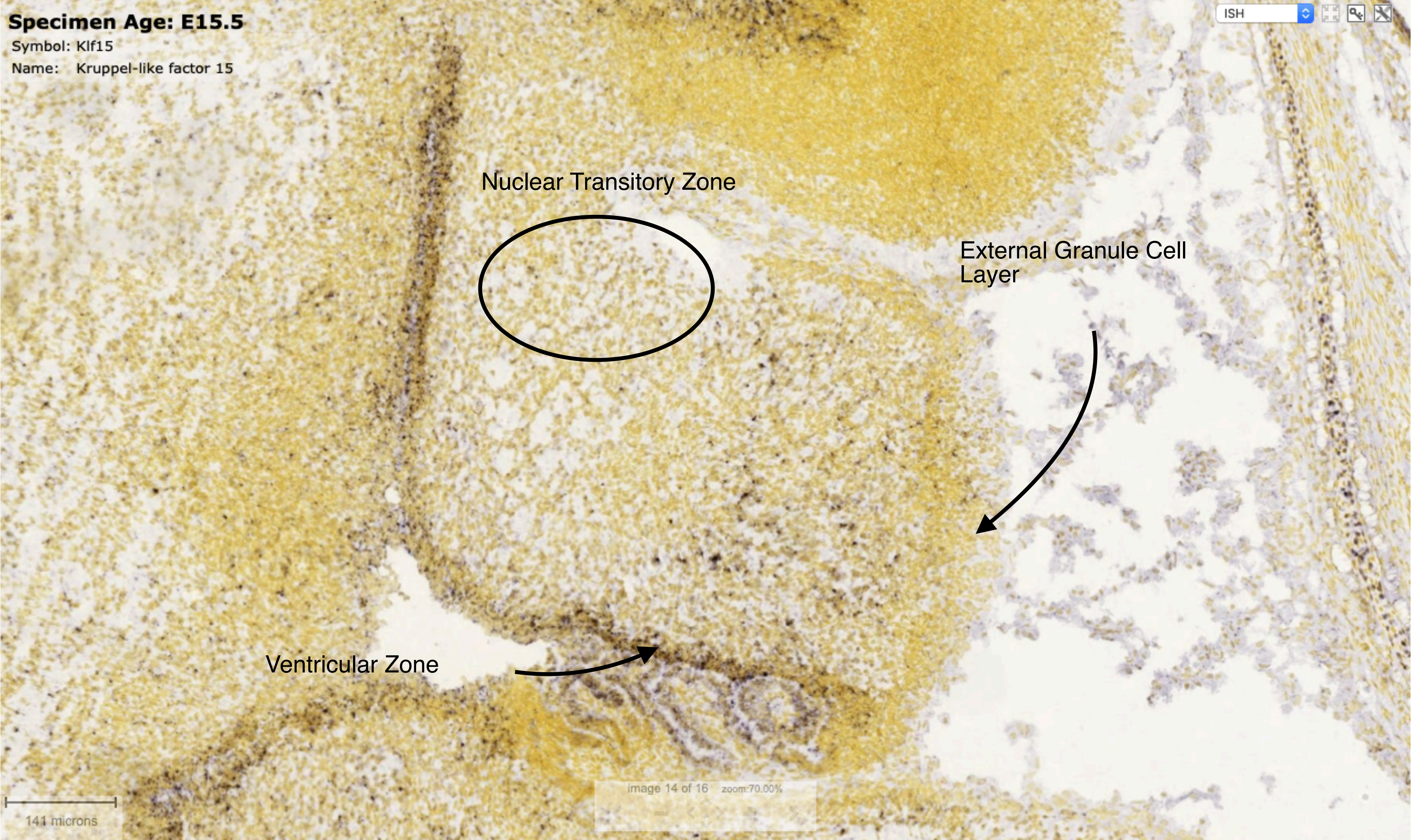
Specimen Age: E15.5
Symbol: Klf15
Name: Kruppel-like factor 15
ISH
Nuclear Transitory Zone
External Granule Cell Layer
Ventricular Zone
image 14 of 16
zoom:70.00%
141 microns

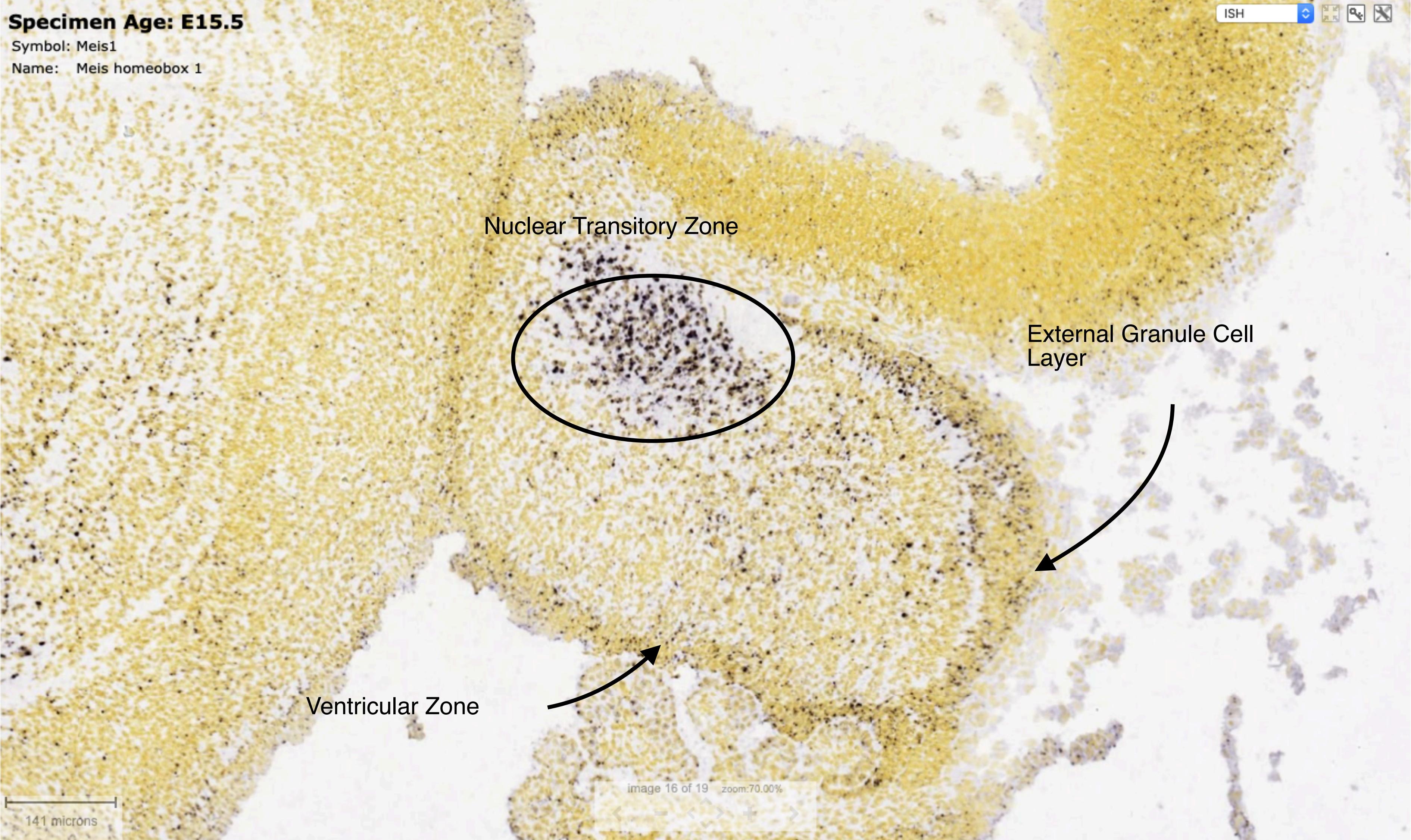

Specimen Age: E15.5
Symbol: Meis1
Name: Meis homeobox 1
ISH
Nuclear Transitory Zone
External Granule Cell Layer
Ventricular Zone
image 16 of 19
zoom:70.00%
141 microns

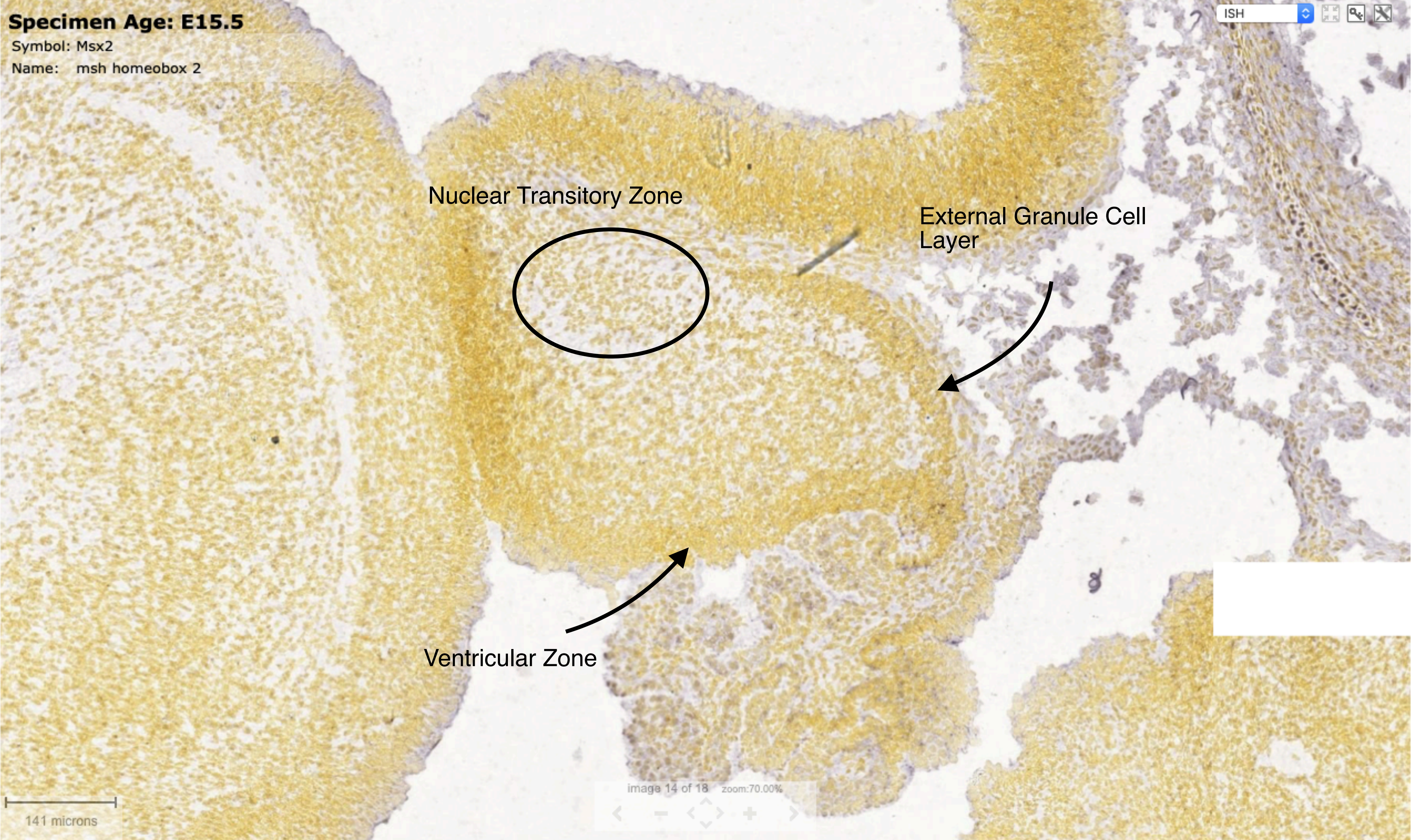
Specimen Age: E15.5
Symbol: Msx2
Name: msh homeobox 2
ISH
Nuclear Transitory Zone
External Granule Cell Layer
Ventricular Zone
image 14 of 18
zoom:70.00%
141 microns

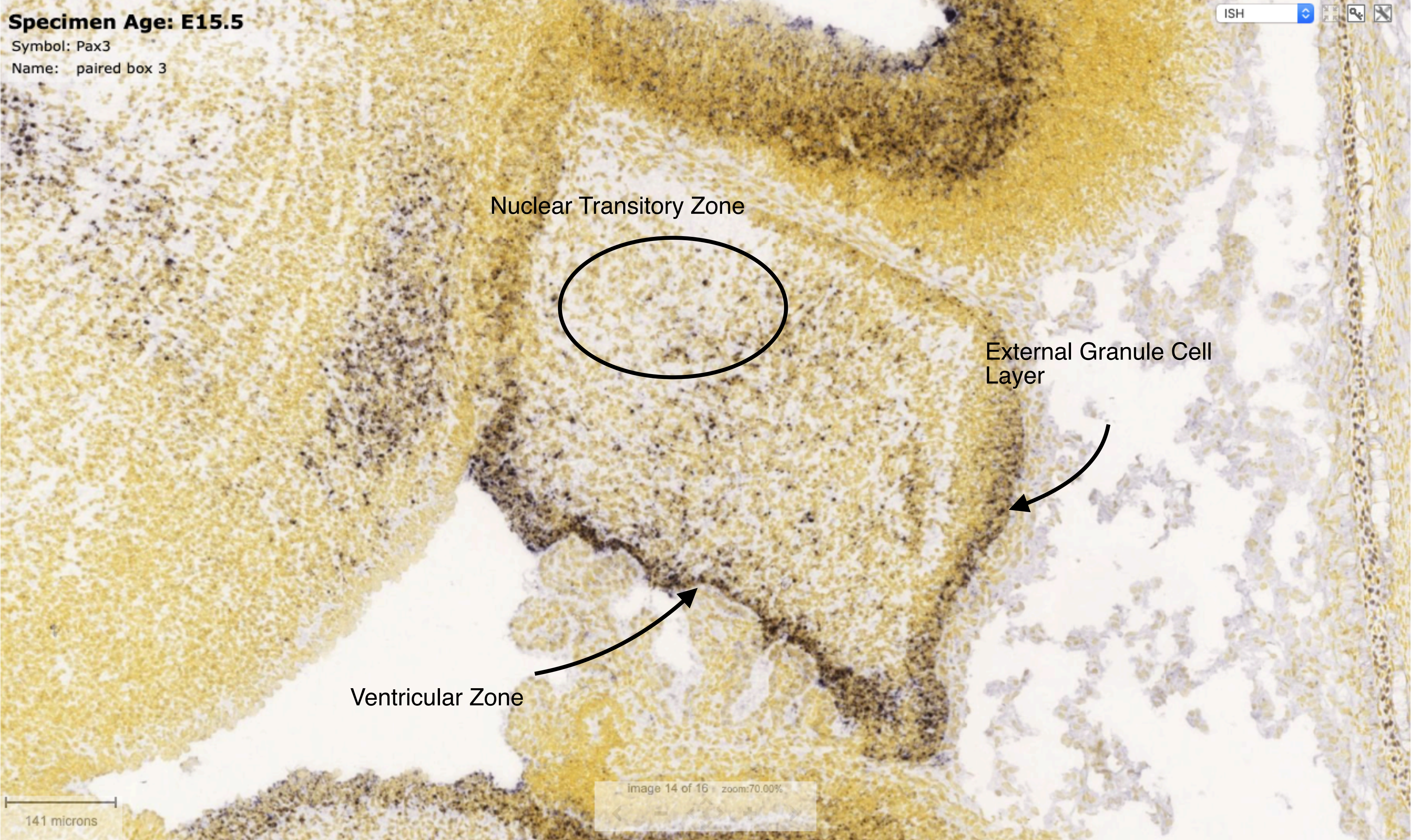
Specimen Age: E15.5
Symbol: Pax3
Name: paired box 3
ISH
Nuclear Transitory Zone
External Granule Cell Layer
Ventricular Zone
image 14 of 16
zoom:70.00%
141 microns

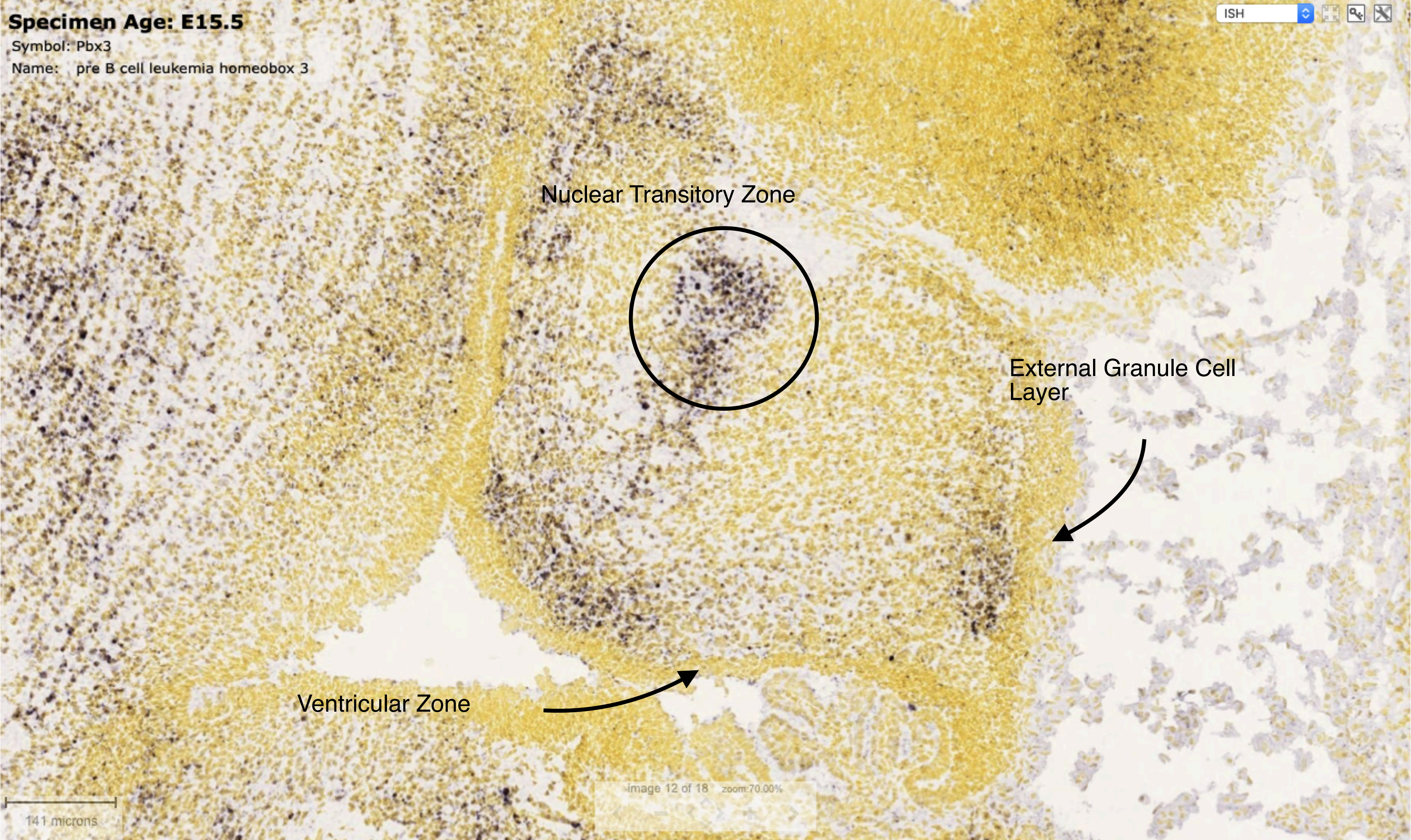
Specimen Age: E15.5
Symbol: Pbx3
Name: pre B cell leukemia homeobox 3
ISH
Nuclear Transitory Zone
External Granule Cell Layer
Ventricular Zone
image 12 of 18
zoom:70.00%
141 microns
