# Supplementary figures and images for: In silico analysis identifies a putative cell-of-origin for BRAF fusion-positive cerebellar pilocytic astrocytoma
Source: PLoS One. 2020 Nov 18;15(11):e0242521. doi: 10.1371/journal.pone.0242521 (PMC7673500; doi:10.1371/journal.pone.0242521)

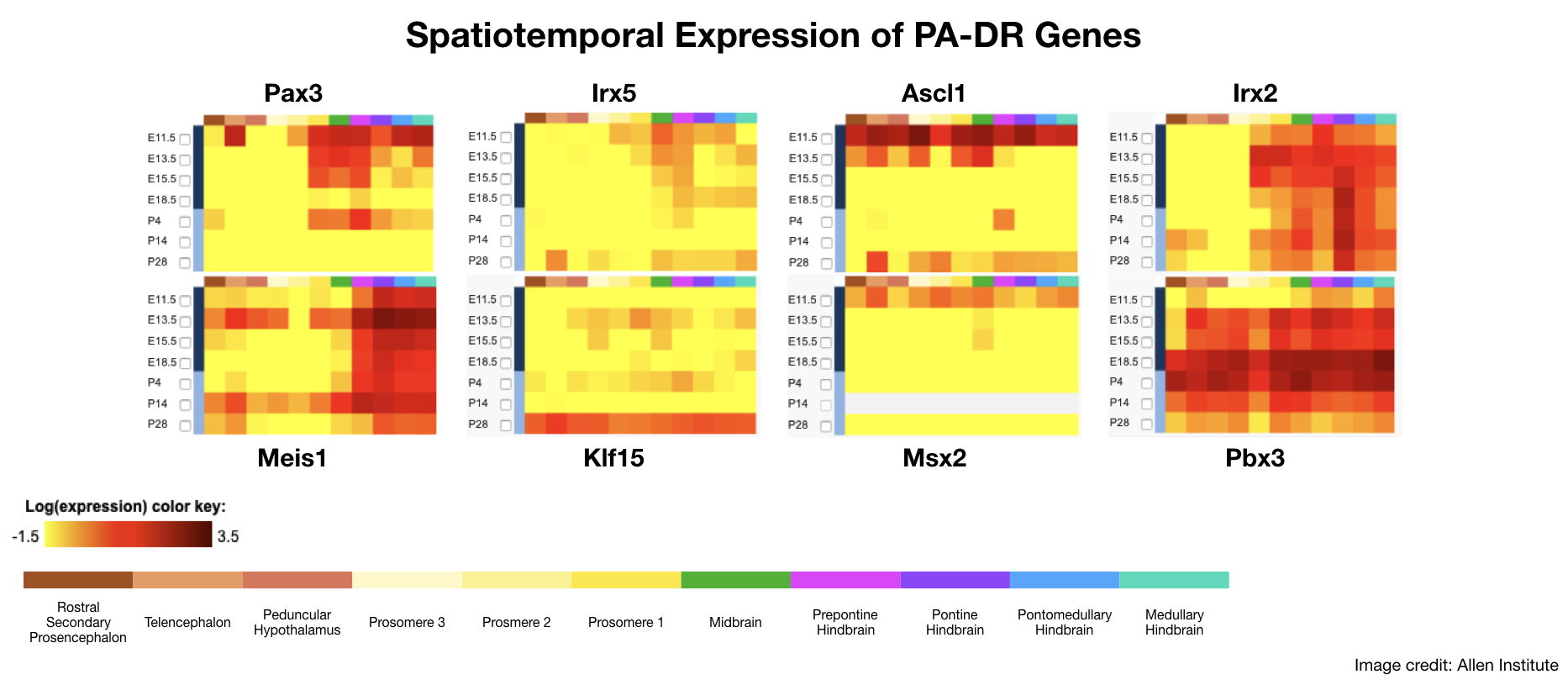

Supplement: S1 Fig — Expression for each PA-DR gene in the Allen Developing Mouse Brain Atlas. Note the particular enrichment on embryonic days 13.5 and 15.5 for all genes. (TIFF) [file pone.0242521.s004.tiff]

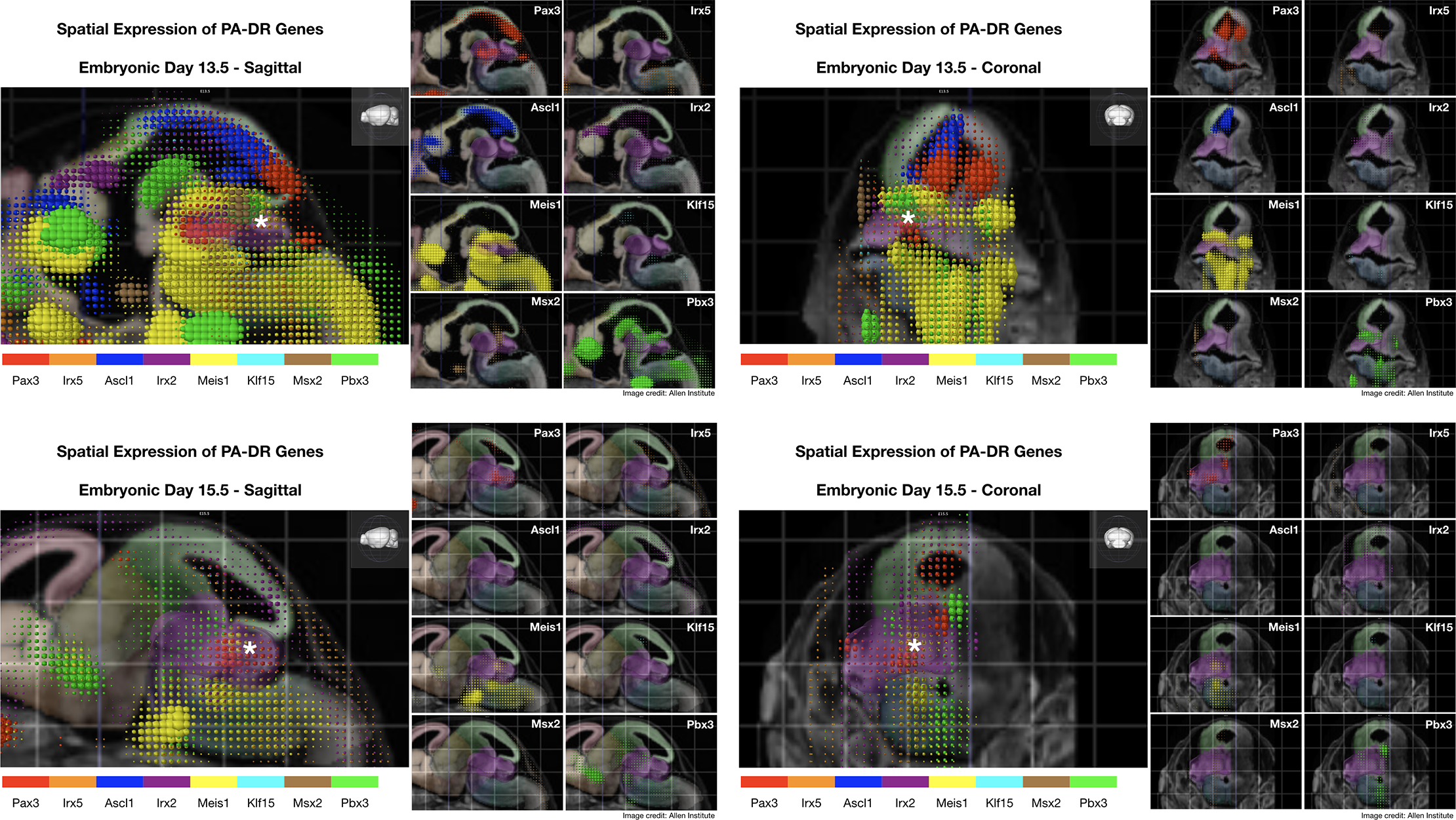

Supplement: S2 Fig — (A-D) Saggital and coronal images of the developing mouse brain with individual gene expression as marked. The purple highlight marks rhombomere 1. The white asterisk marks the cerebellar anlage. Many PA-DR genes exhibit a morphogenic gradient. Six out of eight PA-DR genes are expressed within rhombomere 1. Four are also expressed rostrally and five are also expressed caudally, suggesting rhombomere 1 as the region of overlap for these morphogenic gradients. (TIFF) [file pone.0242521.s005.tiff]

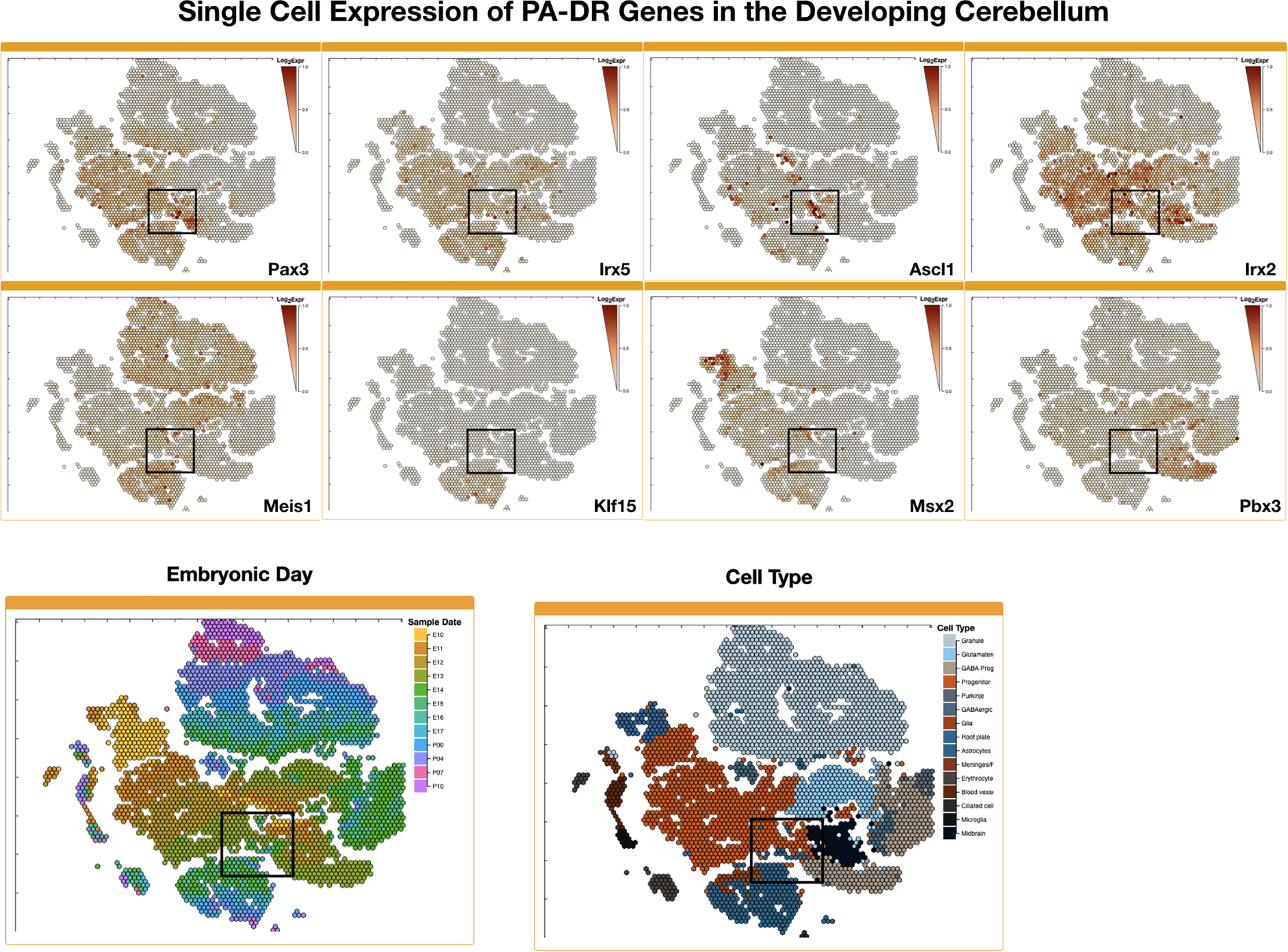

Supplement: S3 Fig — (A) t-SNE plots of single cell gene expression data as in Fig 3A for each PA-DR gene. (B) Developmental day of isolation for single cells shown in Fig 3A. The box denotes the region of overlap of top four PA-DR genes, showing these cells are isolated from embryonic days 13–15. (C) Cell Seek derived cell type for single cells shown in Fig 3A. Based on expression of known cellular markers, cells co-expressing PA-DR genes are identified as early ventricular zone progenitor cells, GABA-ergic neurons, glia, and astrocytes. (TIFF) [file pone.0242521.s006.tiff]

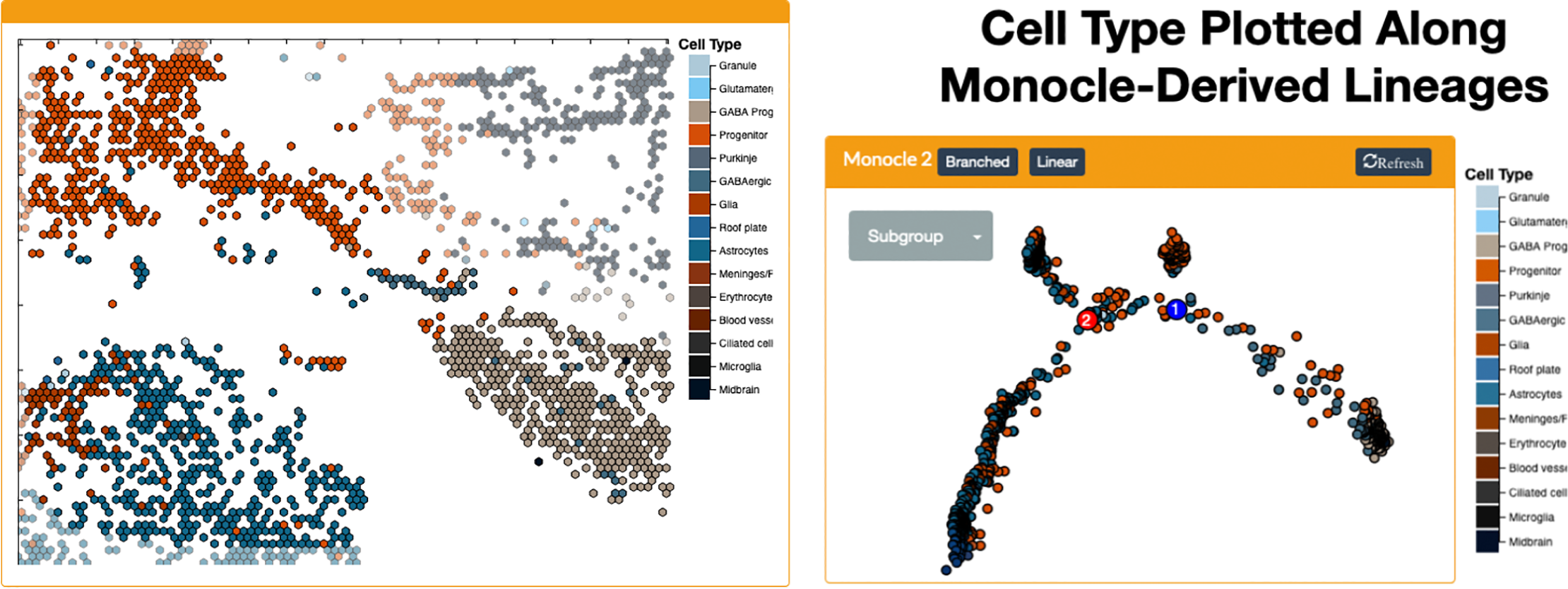

Supplement: S4 Fig — (A) Selection of cells used for subsequent lineage analysis. Bolded hexagons indicate cells which were selected while grayed out hexagons indicate cells which were excluded. (B) Cell seek derived cell types plotted along Monocle derived lineages revealing three main cell types are derived from early ventricular zone progenitor cells: GABA-ergic neuronal progenitors, glial precursor cells, and astrocytes. (TIFF) [file pone.0242521.s007.tiff]

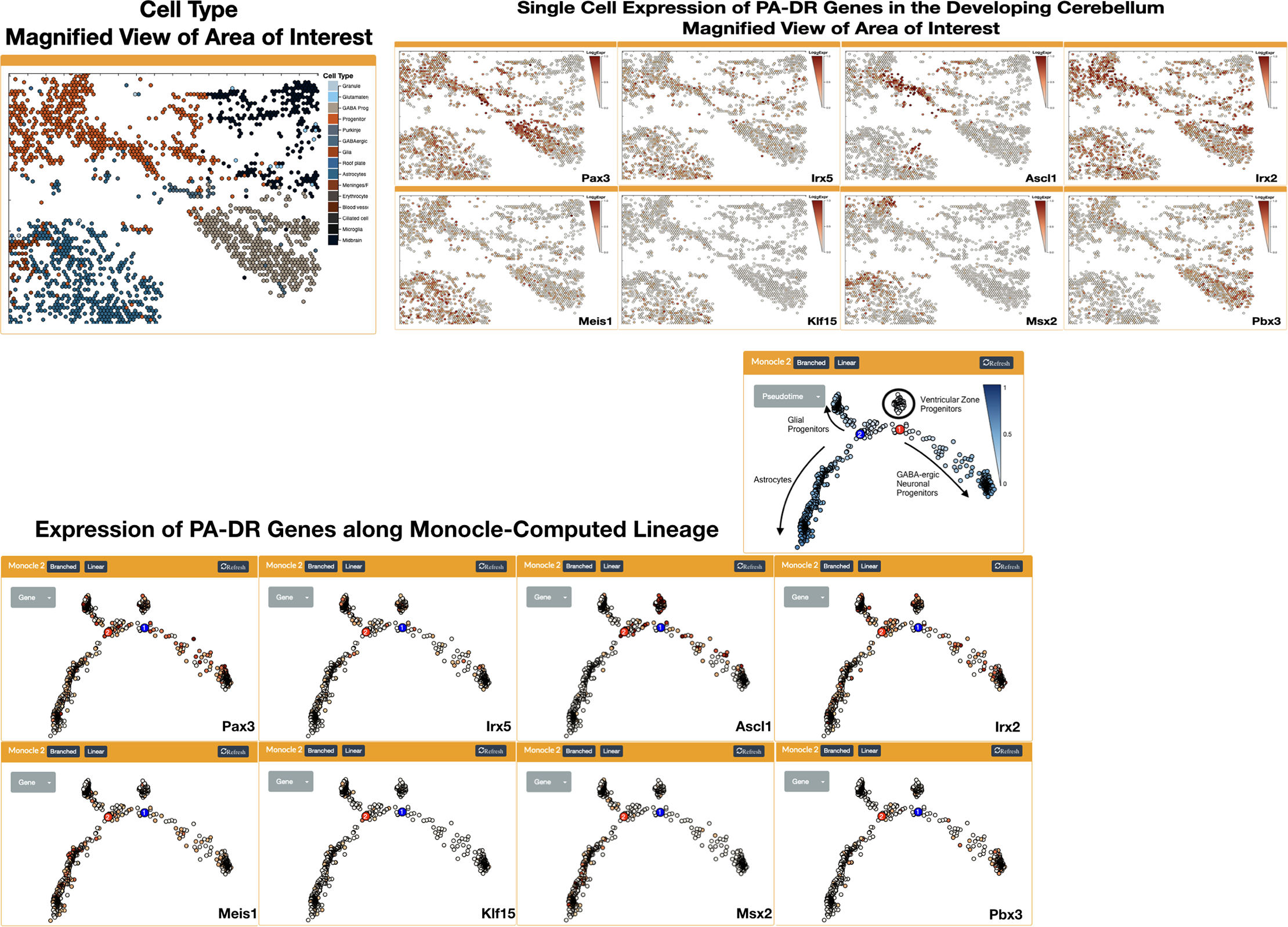

Supplement: S5 Fig — (A) Zoomed region of interest from Fig 3A showing cell type for those cells with strongest overlap in expression of PA-DR genes. (B) Individual PA-DR gene expression for region of interest. Note the temporal relationship and lineage-specific expression of each PA-DR gene (C) Expression data for each PA-DR gene is shown along the Monocle-derived lineages. Note the enrichment of Pax3, Irx5, and Irx2 along all lineages. Ascl1 is enriched for early ventricular zone progenitor cells. Meis1, Klf15, and Msx2 are enriched along the glial progenitor and astrocytic lineages. Pbx3 is expressed chiefly in GABA-ergic neuron progenitor cells. (TIFF) [file pone.0242521.s008.tiff]

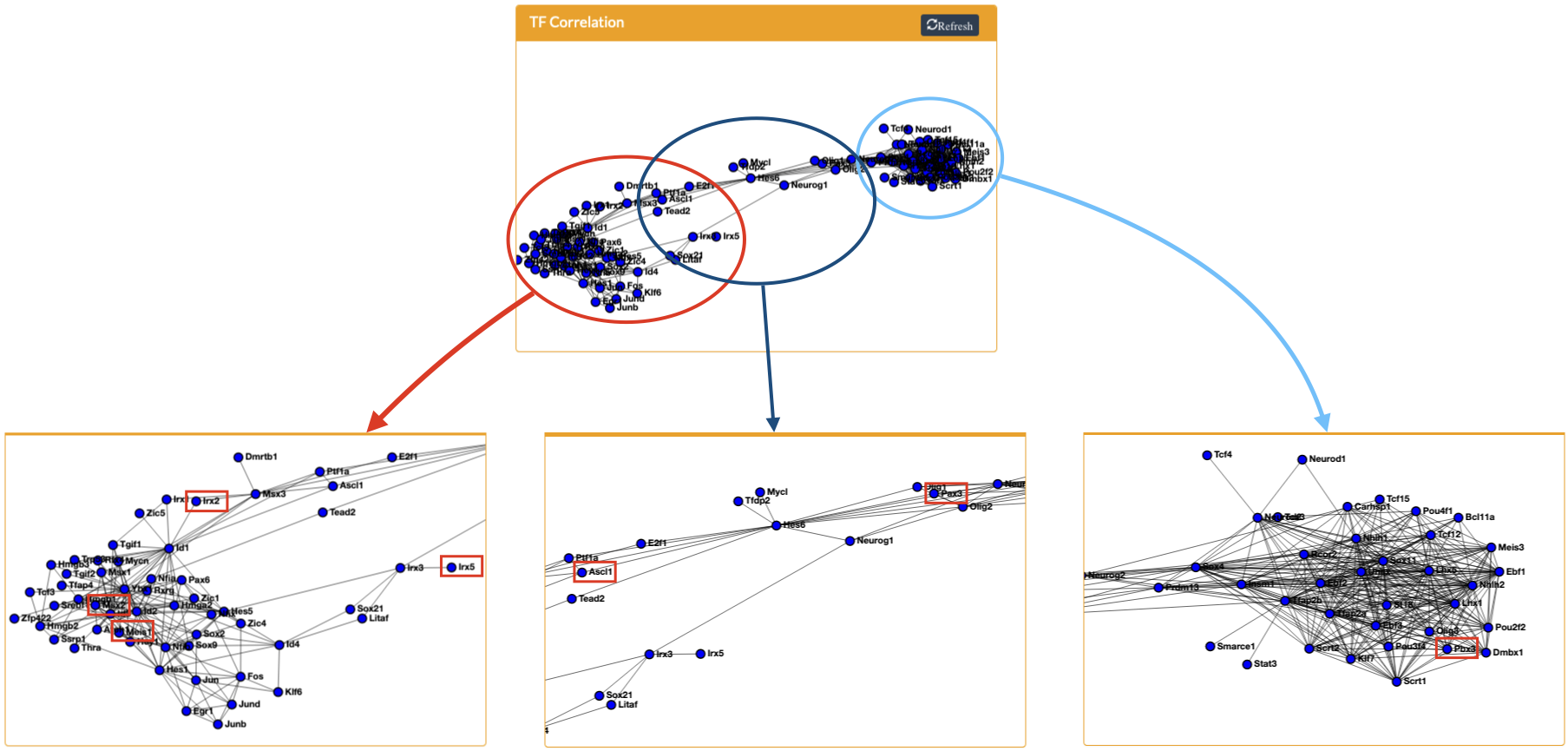

Supplement: S6 Fig — Note that seven out of eight PA-DR genes are represented within the transcription factor network and localization therein recapitulates expression patterns/cell lineage restriction shown in S5 Fig. (TIFF) [file pone.0242521.s009.tiff]
