# Supplementary material for: In silico analysis identifies a putative cell-of-origin for BRAF fusion-positive cerebellar pilocytic astrocytoma
Source: PLoS One. 2020 Nov 18;15(11):e0242521. doi: 10.1371/journal.pone.0242521 (PMC7673500; doi:10.1371/journal.pone.0242521)
Supplement: Attachment — Submitted filename: Response to Reviewers.docx [file pone.0242521.s010.docx]

**Response to Reviewers**

We would like to thank the reviewer and the editor for their thoughtful review and suggestions for improvement. In response, the manuscript has been extensively revised to incorporate the aforementioned reviewer and editor comments.

**Editor Comments**

Comment: The paper could use further review by a biostatistician, as the methodology used is complex.
Response: The methodology has been reviewed by Dr. Kurt Showmaker, a bioinformatician with expertise in differential gene expression analyses. His comments have been used to revise the description of the methodology used.

Comment: I would also advise you to ask a further review by a senior colleague that could give further input.

Response: Dr. Betty Herrington, a pediatric neuro-oncologist, has carefully reviewed the manuscript and analyses. Based on her contributions, she has been added as an author on the manuscript.

**Reviewer Comments**

Comment: In this study, Younes investigates the cell or origin for pediatric cerebellar juvenile pilocytic astrocytoma through an *in silico* approach. This is an ambitious study and carries relevance for the study of pediatric low-grade glioma. However, the study and especially the figures are often confusing and challenging to interpret. The lack of in vitro or in vivo validation of the findings is also a major issue.

Response: The figures and accompanying text have been extensively revised in order to make their interpretation clearer. In particular, the figure legends have been expanded to more carefully walk the reader through the data presented. Regarding the lack of *in vitro* or *in vivo* validation, the discussion has been updated to address these potential shortcomings. Most notably, potential avenues of *in vivo* validation have been proposed.

Comment: Line 37 should say radiation or chemotherapy, since both would rarely be necessary in these tumors.

Response: The line in question has been updated to correctly state that radiation or chemotherapy would be used for the treatment of pilocytic astrocytoma.

Comment: Would add a section on the known molecular alterations common to cerebellar pilocytic astrocytoma.

Response: A paragraph commenting on the known molecular alterations in cerebellar pilocytic astrocytoma has been added to the introduction.

Comment: Line 87, “translocation” – does this refer to BRAF fusions?

Response: This line did indeed seek to refer to BRAF fusion. The entire manuscript has been updated to properly state, “*BRAF* fusion.”

Comment: Need to define “PA-DR” at first mention in this section, not in the middle of Results.

Response: A line defining the designation of PA-DR has been added to the methods section

Comment: There needs to be more justification of the use of a mouse atlas.

Response: Several lines have been added explaining why we chose to utilize mouse atlases first. Briefly, as the reviewer suggests in a later comment, gene expression atlases of the developing human brain are sparse. On the other hand, mouse developmental atlases are more readily available with more robust data. Additionally, a section has been added to the discussion to more fully justify the use of a mouse gene expression atlas.

Comment: Line 162: Again, would change to BRAF fusion (throughout manuscript)

Response: All references throughout the manuscript have been changed to read, “*BRAF* fusion.”

Comment: The legend for Figure 2 is unclear – what do the yellow dots represent?

Response: The legend for Figure 2 has been revised to explain what is depicted.

Comment: Figure 3 is also unclear – color gradients need to be better defined, and images need to be labeled; screen captures from analysis programs generally are not good manuscript figure panels without editing to clarify and remove some unnecessary portions (e.g. a “refresh” button).

Response: Figure 3 has been extensively revised. The legend has been expanded and the figures themselves have been annotated to better direct the reader to the key data points.

Comment: Wouldn’t the lack of transcriptional upregulation of MAPK markers within the developmental cerebellum be because the fusion protein deregulating the pathway in tumors is not present?

Response: The results section in question has been revised to address a comment by the editor. Namely, the editor requested that the comment, “data not shown,” be removed from the manuscript. With the removal of that sentence, the reviewer’s comment has been relieved.

Comment: Again, figure 5 is not clear and needs to be better explained in both the figure (via labels) and legend.

Response: Figure 5 has been extensively revised and annotated to better convey the key data points.

Comment: These findings could likely be validated on a protein level through IHC/IF in human tumor samples, which would substantially strengthen the conclusions.

Response: A review of the literature was performed. Protein-level expression data could only be identified for a single PA-DR gene, namely, Pax3, confirming that it is overexpressed in cerebellar pilocytic astrocytoma. Unfortunately, a lack of funding precludes the ability to interrogate other markers.

Comment: Figure 6: While usually figures should be associated with Results, this may be acceptable if allowed by editors; figure again needs to be better explained, however -- what do the colored arrows represent?

Response: The figure legend has been revised.

Comment: More discussion is needed regarding the use of investigating markers from human tumor samples in a mouse brain atlas and then revalidating with a human atlas -- why not just use the human brain atlas throughout? This may be justifiable in terms of these developmental atlases not being available for humans, but this merits discussion.

Response: The discussion has been revised to more clearly state our rationale for having used mice atlases.
