# Supplementary material for: In silico analysis identifies a putative cell-of-origin for BRAF fusion-positive cerebellar pilocytic astrocytoma
Source: PLoS One. 2020 Nov 18;15(11):e0242521. doi: 10.1371/journal.pone.0242521 (PMC7673500; doi:10.1371/journal.pone.0242521)
Supplement: Attachment — Submitted filename: Response to Reviewers R2.docx [file pone.0242521.s011.docx]

**Response to Reviewers**

We would like to thank the reviewer for their thoughtful review and suggestions for improvement. In response, the manuscript has been revised to incorporate the aforementioned reviewer comments.

**Reviewer Comments**

Comment: The authors have done well in revising the manuscript and have addressed the majority of my concerns. While the figure captions are improved, I would still like to see better keys to the color gradients in panels 3C, 3D, and 5A on the figures themselves to assist readers in interpreting these figures; 3C-D could likely also use text and/or arrows on the panels themselves to help with interpretation.

Response: Figures 3C, 3D, and 5A have each been updated to include a color gradient key. In addition, arrows and text descriptions have been added to figures 3C and 3D to aid the reader in interpreting which direction represents which cell lineage.
